# A Facile Synthesis of Core-Shell SiO_2_@Cu-LBMS Nano-Microspheres for Drug Sustained Release Systems

**DOI:** 10.3390/ma12233978

**Published:** 2019-11-30

**Authors:** Hui Wang, Haifeng Yang, Lifang Zhao

**Affiliations:** 1 College of Chemistry and Chemical Engineering, Shaanxi Key Laboratory of Phytochemistry, Baoji University of Arts and Sciences, Baoji 721013, China; wangkejian@163.com (H.W.); zlfzhao@163.com (L.Z.); 2 College of Physics and Optoelectronics Technology, Medical Micro-nano Materials Research Center, Baoji University of Arts and Sciences, Baoji 721016, China

**Keywords:** layered hydroxide cupric benzoate, SiO_2_@Cu-LBMS, sustained release, drug loading capability

## Abstract

A well-dispersed SiO_2_@Layered hydroxide cupric benzoate (SiO_2_@Cu-LBMS) with a hierarchical structure have been synthesized by a facile method. The layered hydroxide cupric benzoate with a structure of layered basic metal salt (Cu-LBMS) was directly deposited on the surface of silica spheres without any blinder. The morphology of the SiO_2_@Cu-LBMS nano-microsphere was observed by SEM, and the reaction conditions was also discussed. In addition, the XRD patterns and FTIR spectra provide consistent evidence to the formation of SiO_2_@Cu-LBMS nano-microspheres. The release behavior and drug loading capability of SiO_2_@Cu-LBMS microspheres were also investigated by using ibuprofen, aspirin and salicylic acid as model drugs. The results indicated that the drug loading capability of SiO_2_@Cu-LBMS nano-microspheres was much larger than layered hydroxide cupric benzoate, and the releasing time was significantly prolonged than layered hydroxide cupric benzoate and their physical mixture.

## 1. Introduction

Drug delivery systems are designed to either alter pharmacokinetics and biodistribution of their associated drugs, or to function as drug reservoirs. This will enhance several crucial properties of “free” drugs, such as improving their solubility, in vivo stability and specificity, and reducing or eliminating tissue damage [1]. During the past several years, many nanomaterials have been used in drug delivery systems, such as mesoporous silica (MS) [2,3,4], mesoporous magnesium carbonate (MMC) [5], highly porous amorphous calcium carbonates (HPACC) [6], polymeric nanoparticles [7], liposomes [8], carbon nanodots (CDs) [9] and brucite-like materials [10,11,12,13,14,15,16,17]. MS, MMC and HPACC are inorganic nanomaterials, and the mechanism of drug loading mainly depends on pore adsorption. MS presents better performance such as high pore volume and large surface area, but the drug loading capacity was relatively lower than other materials and the release of drugs depends mainly on pore size. Wei Guo et al. successfully synthesized konjac oligosaccharide (KOGC) modified mesoporous silica (mSiO_2_@KOGC), and the drug loading capacity was only 81 mg/g [2]. Shicheng Luo et al. investigated the release behavior of metoprolol tartrate (MPT) in mesoporous silica (MS), it was clearly detected that the release of MPT from different MS demonstrated pore size dependent behaviors, and the maximum release percentage of MPT could only achieve 80% [18]. HPACC had a smaller pore volume and less well-defined pore size than MS and proved to be unstable when exposed to humidity [19]. Polymeric nanoparticles and liposomes demonstrated an excellent biocompatibility. Nevertheless, the process of artificial synthesis for these are too complicated [20]. Compared with materials as mentioned above, layer compounds with a brucite-like structure provide a suitable interlayer spacing for drug molecules. It presents good drug loading capacity and drug releasing performance, facile synthesis, stability, biocompatibility and biodegradability [12,13]. Therefore, it is an excellent candidate for drug delivery systems.

Two types of anion-exchangeable layered compounds with a brucite-like structure have been reported. One is layered double hydroxides (LDHs) with a general formula of [M^II^_1−z_M^III^z(OH)_2_](A^m−^)_z/m_·nH_2_O, where M^II^ is a divalent cation such as Mg^2+^, Fe^2+^, Ni^2+^, Zn^2+^, or Co^2+^; M^III^ is a trivalent cation such as Al^3+^, Cr^3+^, Fe^3+^, or Ga^3+^; and A^m−^ is an exchangeable anion such as CO^3^^−^, NO^3^^−^, OH^−^, X^−^, etc. The z is the molecular ratio of M^3+^/(M^2+^ + M^3+^), generally ranging between 0.2 and 0.4 [16,17,21,22,23,24,25,26]. LHDs with highly positive surface charge, low costs and large area have been demonstrated as good adsorbents to effectively remove acidic or negatively charged compounds and are a desirable carrier for drug or biomolecule delivery [27,28,29,30]. The other type is layered basic metal salts (LBMS). It can be represented by a general formula of [M^II^(OH)_2−x_](A^m−^)_x/m_·nH_2_O, where M^II^ is a divalent basic metal cation such as Cu^2+^, Zn^2+^, Ni^2+^, Mn^2+^; the OH^−^ anions on the brucite hydroxide layer are partially replaced by A^m^^–^ anions; and A^m^^–^ anions are anion-exchangeable [31,32.^33^,^34^]. LBMS have captured much attention in recent years due to their excellent regulatable capability, perfect environmental compatibility and remarkable efficiency [31,35].

However, LBMS and LDHs materials prepared by conventional methods usually resulted in the formation of aggregated powders, which would hamper their potential for exploitation and practical application [36]. Therefore, well-dispersed LDHs or LBMS materials with a hierarchical and open structure would be more attractive for the practical application. Chen et al. reported a simple method to fabricate well-dispersed SiO_2_@LDH hierarchical spheres for efficient removal of pharmaceuticals from water [36,37]. Zhu et al. explored a core-shell nanomaterial SiO_2_@LDH and modified SiO_2_@LDH with Bevacizumab (Bev) to form a new tumor vasculature targeting nanocarrier, SiO_2_@LDH-Bev, as vector of DOX (Doxorubicin Hydrochloride). The drug loading capacity was 134.6 mg/g and 205.6 mg/g, respectively [38]. Silicon oxide (SiO_2_) has the characteristics of being non-toxic, non-polluting, small-sized and having good dispersibility, as well as being a good template material for preparing organic and inorganic composites [39,40,41,42,43,44,45,46]. In this article, we reported a facile method of making well-dispersed SiO_2_@Cu-LBMS nano-microspheres which was not reported before.

As carrier materials for drug sustained release systems, many hydrotalcite-like materials are administered orally. For example, Haraketi et al. intercalated salicylic acid into the interlayers of the Cu-AL-LDHs to form an oral sustained release drug delivery system**, **and detected that 3 h was needed to release 90% of entrapped drugs from Cu-AL-LDHs [47]. Pooresmaeil et al. loaded 5-fluorouracil (5-Fu) in the synthesized Zn-Al-LDHs to form a new nanovehicle for oral colorectal cancer treatment [48]. In this paper, the SiO_2_@Cu-LBMS nano-composite microsphere we synthesized by a facile method was used as a carrier material for oral drug sustained release system. We also investigated the outstanding properties of drug loading capacity and drug releasing performance.

## 2. Materials and Methods

### 2.1. Materials

The chemical crude drug ibuprofen, aspirin and salicylic acid were purchased from Adamas Reagent Co, Ltd. (Shanghai, China). All the other starting materials used in this work were of analytical grade and were purchased from Tianjin Kemiou Chemical Reagent Co., Ltd. (Tianjin, China).

### 2.2. Methods

#### 2.2.1. Preparation of Cu-LBMS

2.40 mL ammonia was added dropwise into a 0.32 mol/L Cu(NO)_3_ (50 mL) solution at room temperature under stirring conditions. The mixture was stirred continually for 0.5 h. The product was aged in the reaction solution at room temperature for 6 h, and then was filtered, washed with distilled water, and dried at room temperature for 24 h. This sample was used as the starting material for the preparation of Cu-LBMS.

Cu-LBMS was synthesized according to the references [35,49]. 1.60 g of the starting material and 0.97 g benzoic acid and 25 mL water was added in a three-necked round-bottom flask, stirred reflux 24 h. At the end of the reaction, the product was filtered, washed, and dried at room temperature for 24 h, and the layered hydroxide cupric benzoate (Cu-LBMS) was obtained.

#### 2.2.2. Preparation of SiO_2_ Microspheres

According to the references [50,51], The A solution was prepared by mixed 31.50 mL water, 30.00 mL ammonia water and 20.00 mL ethanol under 30 °C. The B solution was prepared by mixing 50.00 mL ethanol and 11.00 mL ethyl orthosilicate under the same temperature. The B solution was added drop-wise into the A solution, stirring continuously for 0.5 h and aging 24 h. The SiO_2_ microspheres were obtained by centrifuge, washed with distilled water, and then dried at room temperature.

#### 2.2.3. Hybrid Assembly of Cu-LBMS with SiO_2_ Microspheres

0.10 g Cu-LBMS, 0.10 g SiO_2_ microspheres, and 20.0 mL water was put in a three-necked round-bottom flask and then stirred at 95 °C for 24 h. The layered composite nano-microspheres SiO2@Cu-LBMS was obtained by filtrate, washed with distilled water, and then dried at room temperature.

#### 2.2.4. Preparation of the Drug-Loaded SiO_2_@Cu-LBMS and Drug-Loaded Cu-LBMS

For the preparation of the ibuprofen-loaded SiO_2_@Cu-LBMS, the ibuprofen aqueous solution (5.00 mg ibuprofen was dissolved in 50 mL ethanol) and 10.00 mg SiO_2_@Cu-LBMS was put in a 100 mL beaker and stirred for 2 h at 60 °C, and then incubated for 24 h at 60 °C. The ibuprofen-loaded SiO_2_@Cu-LBMS was obtained by filter and washed with distilled water. The aspirin-loaded SiO_2_@Cu-LBMS and the salicylic acid-loaded SiO_2_@Cu-LBMS was prepared by the same method. The drug loading capacity of SiO_2_@Cu-LBMS was calculated using Equation (1)

Drug loading capacity = W_C_/W (1)

where W_C_ represents the weights of the loaded drug in SiO_2_@Cu-LBMS; and W represents the weights of drug-loaded SiO_2_@Cu-LBMS.

The drug-loaded Cu-LBMS was prepared by the same methods and the drug loading capacity of Cu-LBMS was also calculated using Equation (1).

#### 2.2.5. In vitro Drug Cumulative Release Studies

The in vitro ibuprofen release behaviors from SiO_2_@Cu-LBMS were carried out in a dissolution apparatus (Huanghai RCZ-1B). A weighed amount of the sample was immersed in 500 mL of dissolution media and stirred at 50 rpm at 37 °C ± 0.5 °C (in pH 7.4 PBS). At certain time intervals, 4.0 mL of the sample was collected from the suspension and replaced with the same amount of the fresh dissolution media. Then, the mixture (4.0 mL) centrifuged at a rotation rate of 10,000 rpm for 10 min to obtain the supernatant. The cumulative amount of ibuprofen released from Cu-LBMS/SiO_2_@Cu-LBMS can be determined using a UV-vis spectrophotometer (MAPADA) at 264 nm. The in vitro aspirin and salicylic acid release behaviors from SiO_2_@Cu-LBMS were carried out according to the same procedure except that aspirin was determined at 300 nm and salicylic acid was determined at 280 nm. The drug cumulative release percent was calculated using Equation (2).

Drug cumulative release percent (%) = M_t_/M × 100% (2)

M_t_ represents the cumulative amount of drugs released at time t; and M represents the initial amount of drugs loaded. The in vitro drug release studies were carried out in triplicate and the average values were shown. The in vitro drug release behaviors from Cu-LBMS and the release of drugs from the physical mixture were studied in the same method and also calculated using Equation (2). 

#### 2.2.6. Characterization

FTIR spectra of the samples were carried out on a PerkinElmer Frontier FT-IR spectrometer (PerkinElmer, MA, USA). Powder X-ray diffraction (XRD) analysis of the specimens was performed using an X-ray power diffractometer with Cu anode (Rigaku, DMAX U1TIMA IV, Tokyo, Japan), running at 40 kV and 40 mA, scanning from 3° to 60°. Surface morphology of the nano-microspheres was investigated by scanning electron microscopy (SEM, FEI, Quanta 250EFG, Oregon, USA). Before observation, all the samples were placed on round brass stubs and sputter-coated with platinum under an argon atmosphere. The data of the particle size and its distribution were measured by software “Nano Measure”.

## 3. Results and Discussion

### 3.1. The Morphology of SiO_2_@Cu-LBMS

**Figure 1:**
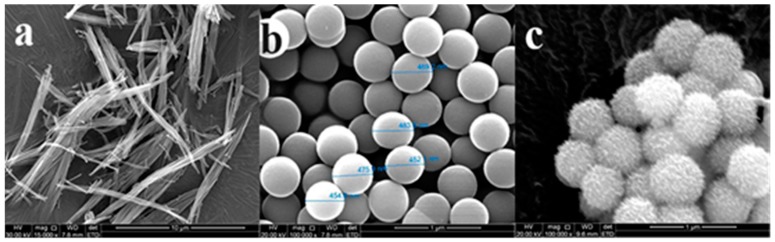
SEM micrographs of (**a**) Cu-LBMS; (**b**) SiO_2_; and (**c**) SiO_2_@Cu-LBMS.

The interaction of Cu-LBMS and SiO_2_ microspheres may change the surface morphology of SiO_2_ microspheres, which has great influence on the drug release behaviors. Thus, the surface morphology of dried Cu-LBMS, SiO_2_ microspheres and SiO_2_@Cu-LBMS were observed by SEM. The SEM image in Figure 1a shows that the surface morphology of layered hydroxide cupric benzoate (Cu-LBMS) are fibrous. Figure 1b shows that SiO_2_ spheres prepared in this work are monodispersed with a uniform diameter of around 450 nm (the statistics of size as shown in Figure S1a). The surface of SiO_2_ spheres is negatively charged in a neutral environment as indicated in this work. Hence, Cu-LBMS can be deposited directly on silica by electrostatic attraction. After coating with the Cu-LBMS layer, the surface of the sample becomes loose porous spheres which indicates that a uniform layer of Cu-LBMS nanosheets homogenously grows from the surface of the silica spheres. It can also be observed that the final composite sample consists of well-dispersed spheres with an average diameter of around 600 nm (the statistics of size as shown in Figure S1b).

### 3.2. Hybrid Assembly Process of Cu-LBMS and SiO_2_

**Figure 2:**
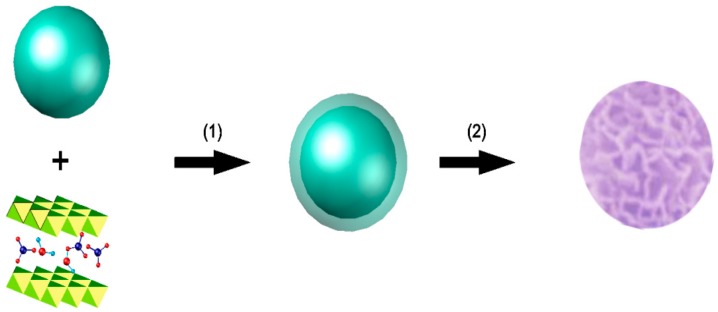
Schematic illustration of the hybrid assembly process of Cu-LBMS and SiO_2._

As shown in Figure 2, the hybrid assembly route of Cu-LBMS on silica spheres was performed via two steps: step 1, precipitation and step 2, in situ growth. Cu-LBMS was directly deposited on the surface of silica sphere without any binder. In this work, the silica particles carry a negative charge in aqueous solutions at pH 7, and this provides an excellent substrate for positively-charged Cu-LBMS precursors to be electrostatically attracted to the silica surface. We suspect that the formation of Cu-LBMS nanosheets on the surface of the silica spheres is a result of electrostatic interactions, molecular interaction and hydrogen-bond interaction.

### 3.3. Preparation of SiO_2_@Cu-LBMS Nanocomposite

**Figure 3:**
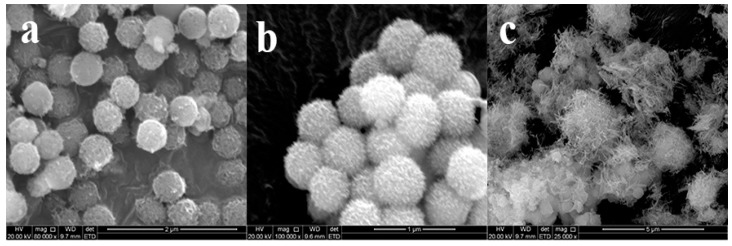
SEM micrographs of SiO_2_@Cu-LBMS with varying ratios of Cu-LBMS:SiO_2_ (**a**) 1:2; (**b**) 1:1; and (**c**) 2:1.

It seems that three factors played major roles on the morphology of SiO2@Cu-LBMS nanocomposite in this work: (i) mass ratio of Cu-LBMS:SiO_2_; (ii) reaction temperature; (iii) reaction time. To verify this, several sets of controlled samples were prepared. Different SiO_2_@Cu-LBMS samples with varied morphologies were obtained. The first set of controlled samples were prepared by using the same reaction condition (reaction temperature 95 °C, reaction time 24 h) but varying the mass ratio of Cu-LBMS:SiO_2_. Figure 3 shows the SEM micrographs of SiO_2_@Cu-LBMS with varying ratios of Cu-LBMS:SiO_2_. The images clearly show that Cu-LBMS nanosheets deposit on the surface of the silica spheres. Additionally, it is interesting to observe that some of the SiO_2_ were not covered with Cu-LBMS when Cu-LBMS:SiO_2_ mass ratio was 1:2 (Figure 3a). However, when the ratio increased to 2:1, a larger and denser Cu-LBMS was formed on the surface of SiO_2_, as shown in Figure 3c, and irregular shapes were obtained. When the ratio was 1:1, loose, porous and regular microspheres were obtained, as shown in Figure 3b.

**Figure 4:**
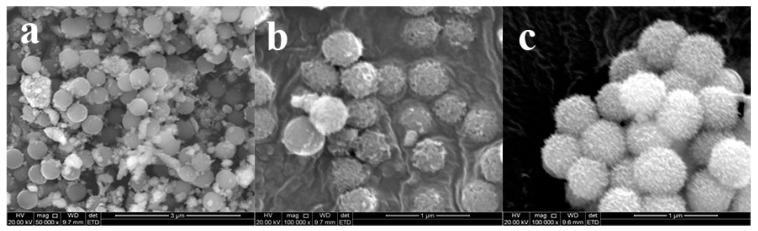
SEM micrographs of SiO_2_@Cu-LBMS with different reaction temperature (**a**) 50 °C; (**b**) 60 °C; and (**c**) 95 °C.

The second set of controlled samples were prepared by using the same reaction condition (the mass ratio of Cu-LBMS:SiO_2_ 1:1, reaction time 24 h) but varying the reaction temperature. The reaction was carried out at 50 °C, 60 °C and 95 °C, respectively, and different SiO_2_@Cu-LBMS samples were obtained. As shown in Figure 4, most of the SiO_2_ were not covered with Cu-LBMS when the reaction temperature was 50 °C (Figure 4a), and when temperature was 60 °C, incomplete reaction of SiO_2_ and Cu-LBMS were observed, as shown in Figure 4b. However, loose, porous and regular microspheres of SiO_2_@Cu-LBMS samples were obtained when reaction temperature was 95 °C.

**Figure 5:**
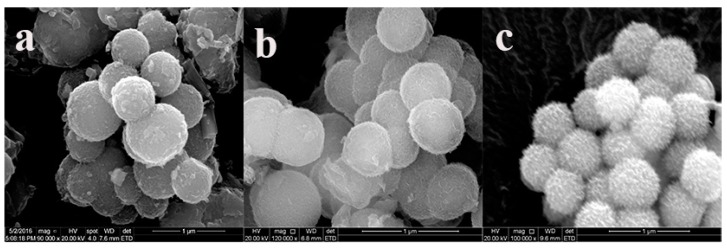
SEM micrographs of SiO_2_@Cu-LBMS with different reaction time (**a**) 5 h; (**b**) 12 h; and (**c**) 24 h. We also compared the effect of reaction time 19 h with 37 h on the morphology of SiO_2_@Cu-LBMS nanocomposite. When the reaction time was 19 h, some well-dispersed spheres with porous surface were formed; however, there were still some SiO_2_ spheres not covered by porous structure, as shown in Figure S2a. When the reaction time was extended to 37 h, uneven Cu-LBMS layers were formed on the surface of SiO_2_ spheres, and further aggregates can be observed for this sample, as shown in Figure S2b.

The third set of controlled samples were prepared by using the same reaction condition (the mass ratio of Cu-LBMS:SiO_2_ 1:1, reaction temperature 95 °C) but varying the reaction time. We found that the reaction time also played a major role on the morphology of SiO_2_@Cu-LBMS nanocomposite in this work. When the reaction time was 5 h, Cu-LBMS were randomly but densely packed on the surface of SiO_2_ spheres, as shown in Figure 5a. When the reaction time increased to 12 h, some Cu-LBMS started growing vertically on the surface of SiO_2_, as shown in Figure 5b. Figure 5c revealed that the final composite sample consists of well-dispersed spheres with loose porous surface which were obtained as the reaction time was extended to 24 h.

### 3.4. The Physicochemical Properties of SiO_2_@Cu-LBMS Microspheres

**Figure 6:**
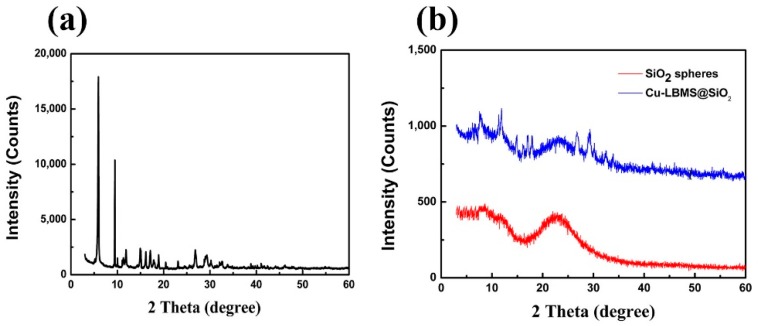
XRD patterns of (**a**) Cu-LBMS; and (**b**) SiO_2_ spheres and SiO_2_@Cu-LBMS.

**Figure 7:**
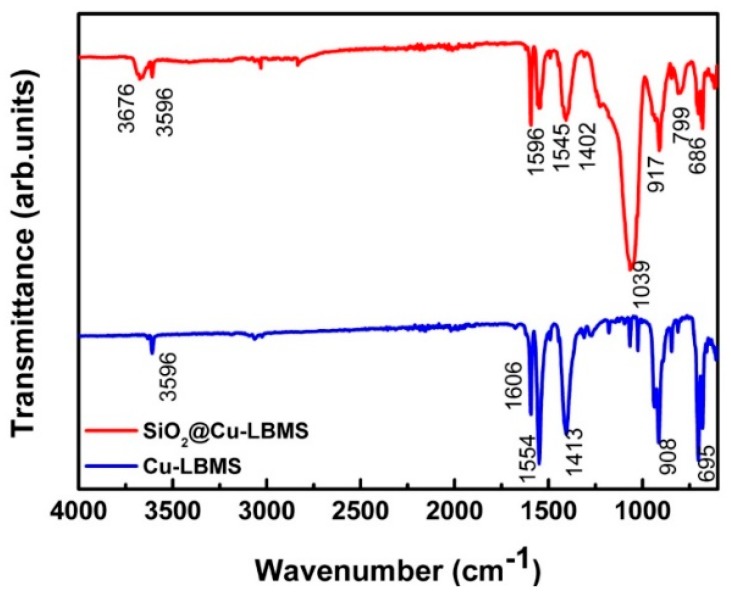
FTIR spectra of SiO_2_@Cu-LBMS and Cu-LBMS.

The physicochemical properties of SiO_2_@Cu-LBMS were characterized by XRD and FTIR. As shown in Figure 6 and Figure 7, the XRD patterns and FTIR spectra provide consistent evidence to the formation of SiO_2_@Cu-LBMS microspheres.

The XRD data in Figure 6a shows the characteristic diffraction peaks of Cu-LBMS synthesized in our work (consisting of typical layer features with a series of diffraction peaks) [32]. Figure 6b indicates that the SiO_2_ spheres have a common amorphous structure [50,52]. After in situ growth of Cu-LBMS nanosheets on the silica spheres, the XRD of SiO_2_@Cu-LBMS exhibits features that are a combination of both Cu-LBMS and silica spheres.

In the FTIR spectrum of Cu-LBMS, the strong absorption bands at 1554 cm^−1^ and 1413 cm^−1^ (asymmetric and symmetric stretching vibration of –COO^−^ groups), the absorption bands at 3596 cm^−1^ (O–H stretching vibration of Cu–OH), 1606 cm^−1^ (C=C stretching vibration of benzene skeleton), 695 cm^−1^ (C–H bending vibration of benzene), 908 cm^−1^ (stretching vibration of Cu–O) are observed, which are the characteristic absorption bands of Cu-LBMS. After the combination of Cu-LBMS and SiO_2_, the absorption bands of SiO_2_ at 1039 cm^−1^and 799 cm^−1^ (asymmetric and symmetric stretching vibration of Si–O) were observed, and the absorption bands of Cu-LBMS at 1606 cm^−1^, 1554 cm^−1^, 1413 cm^−1^,695 cm^−1^ and 908 cm^−1^ were obviously weakened and shifted to 1596 cm^−1^, 1545 cm^−1^, 1402 cm^−1^, 686 cm^−1^ and 917 cm^−1^.

### 3.5. The Drug Loading Property of SiO_2_@Cu-LBMS Microspheres

To demonstrate the potential application of SiO_2_@Cu-LBMS microspheres, the drug loading property of the microspheres was studied by using ibuprofen, aspirin and salicylic acid as model drugs. As can be seen, the drug loading of SiO_2_@Cu-LBMS for ibuprofen, aspirin and salicylic acid was respectively 263.16 mg/g, 380.14 mg/g and 362.23 mg/g, whereas the drug loading of Cu-LBMS for this drug was respectively 160.05 mg/g, 300.02 mg/g and 254.13 mg/g. The drug loading capacity of SiO_2_@Cu-LBMS was much higher than Cu-LBMS and some nanomaterials, as mentioned in the introduction [2,38]. We have reported the structure of layered basic metal salts (LBMS) [32], in which basic metal ions are in polyhedral sites in the brucite hydroxide layer and benzoic acid anions are in the interlayer space. This structure lead to the absorption of drugs into the interlayer space. When Cu-LBMS were assembled with SiO_2_ microspheres, the morphology of SiO_2_@Cu-LBMS microspheres are changed to porous spherical. As can be seen from Table 1, the SiO_2_@Cu-LBMS microspheres exhibit much enhanced drug loading capacity compared to Cu-LBMS. In our SiO_2_@Cu-LBMS microsphere, individual Cu-LBMS nanosheets are more likely accessible to the drug molecules probably due to the good dispersion of the nanosheets. The relatively lower drug loading performance of Cu-LBMS was probably due to the serious agglomeration of the constituent crystals.

### 3.6. The Effects of SiO_2_@Cu-LBMS Microspheres on Drug Release Behavior

**Figure 8:**
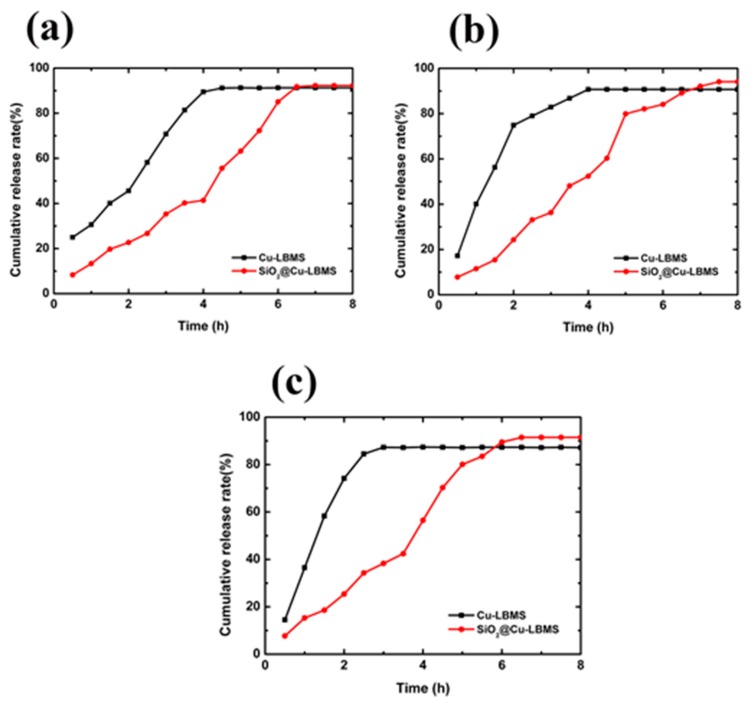
Cumulative release of (**a**) ibuprofen; (**b**) aspirin; and (**c**) salicylic acid.

The effects of Cu-LBMS and SiO_2_@Cu-LBMS microspheres on drug release behavior were investigated, also by using ibuprofen, aspirin and salicylic acid as model drugs. Figure 8a and Table S1 demonstrates that 91% of entrapped ibuprofen were released from Cu-LBMS after being maintained in simulated body fluid for 4.5 h. Whereas, approximately 7 h was needed for SiO_2_@Cu-LBMS microspheres to release 91% of entrapped ibuprofen. The release of ibuprofen from the physical mixture was already 100% when left in simulated body fluid for less than 30 min (Figure S3). At the same time point (30 min), 25% of entrapped ibuprofen were released from Cu-LBMS and only 8.3% of entrapped ibuprofen were released from SiO_2_@Cu-LBMS microspheres. The results make a distinct conclusion that the releasing time of SiO_2_@Cu-LBMS for ibuprofen is obviously prolonged than that of Cu-LBMS and the physical mixture. 

Beside ibuprofen, the sustained release effect of SiO_2_@Cu-LBMS on other drugs is also much better than that of Cu-LBMS and their physical mixture, such as aspirin and salicylic acid. The major release process of aspirin and salicylic acid from Cu-LBMS took place within 4 h and 3.5 h respectively. Whereas, 7.5 h was needed for SiO_2_@Cu-LBMS microspheres to release 94.1% of entrapped aspirin (Figure 8b and Table S1) and 6.5h was needed for SiO_2_@Cu-LBMS microspheres to release 91.5% of entrapped salicylic acid (Figure 8c and Table S1). The release of aspirin and salicylic acid from the physical mixture was already 100% after 25 min (Figure S3). In addition, the sustained release effect, such as maximum cumulative release rate and releasing time of SiO_2_@Cu-LBMS, is obviously better than some materials, as mentioned in the introduction [18,47].

## 4. Conclusions

In this paper, SiO_2_@Cu-LBMS were successfully prepared by a facile method and characterized by SEM, XRD and FTIR. The morphology of SiO_2_@Cu-LBMS were porous microspheres as observed by SEM, and the XRD patterns and FTIR spectra also evidently demonstrated the formation of SiO_2_@Cu-LBMS microspheres. The layered hydroxide cupric benzoate with structure of layered basic metal salt (Cu-LBMS) was uniformly deposited on the surface of silica spheres when the mass ratio of Cu-LBMS:SiO_2_ was 1:1, the reaction temperature was 95 ℃ and the reaction time was 24 h. The as-prepared SiO_2_@Cu-LBMS microsphere exhibits excellent drug loading capability and drug release performance by using ibuprofen, aspirin and salicylic acid as model drugs. Hence, our works demonstrate a facile method for synthesis of core-shell SiO_2_@Cu-LBMS nano-microspheres which have broad application potential for drug sustained release systems. It is expected that the new material SiO_2_@Cu-LBMS microspheres fabricated and the method used in this work provides new insights into designing functional layered composite nano-microspheres for a wide range of applications.

## Figures and Tables

**Table 1:** The drug loading capacity of Cu-LBMS and SiO_2_@Cu-LBMS.

Materials	Ibuprofen (mg/g)	Aspirin (mg/g)	Salicylic Acid(mg/g)
SiO_2_@Cu-LBMS	263.16	380.14	362.23
Cu-LBMS	160.05	300.02	254.13

## Supplementary Materials

The following are available online at https://www.mdpi.com/1996-1944/12/23/3978/s1, Figure S1: Particle size distribution of (a) SiO_2_; (b) SiO_2_@Cu-LBMS, Figure S2: SEM micrographs of SiO_2_@Cu-LBMS with different reaction time (a) 19h; (b) 37h, Figure S3: Cumulative release of physical mixture, Table S1: Drug cumulative release rate (%) of Cu-LBMS and SiO_2_@Cu-LBMS.

## References

[1] De M., Ghosh P.S., Rotello V.M. (2008). Applications of Nanoparticles in Biology. Adv. Mater..

[2] Guo W., Yang C., Cui L., Lin H., Qu F. (2014). An Enzyme-Responsive Controlled Release System of Mesoporous Silica Coated with Konjac Oligosaccharide. Langmuir.

[3] Zhang P., Forsgren J., Strømme M. (2014). Stabilisation of amorphous ibuprofen in Upsalite, a mesoporous magnesium carbonate, as an approach to increasing the aqueous solubility of poorly soluble drugs. Int. J. Pharm..

[4] Tian B.-S., Yang C. (2011). Thermo-Sensitive Poly(*N*-Isopropylacrylamide)/Mesoporous Silica Nanocomposites as Controlled Delivery Carriers: Loading and Release Behaviors for Drug Ibuprofen. J. Nanosci. Nanotechnol..

[5] Zhang P., Zardán Gómez de la Torre T., Forsgren J., Bergström C.A.S., Strømme M. (2016). Diffusion-Controlled Drug Release from the Mesoporous Magnesium Carbonate Upsalite^®^. J. Pharm. Sci..

[6] Trofimov A.D., Ivanova A.A., Zyuzin M.V., Timin A.S. (2018). Porous Inorganic Carriers Based on Silica, Calcium Carbonate and Calcium Phosphate for Controlled/Modulated Drug Delivery: Fresh Outlook and Future Perspectives. Pharmaceutics.

[7] DeLeon V.H., Nguyen T.D., Nar M., D’Souza N.A., Golden T.D. (2012). Polymer nanocomposites for improved drug delivery efficiency. Mater. Chem. Phys..

[8] Wolfram J., Scott B., Boom K., Shen J., Borsoi C., Suri K., Grande R., Fresta M., Celia C., Zhao Y. (2016). Hesperetin Liposomes for Cancer Therapy. Curr. Drug Deliv..

[9] Scialabba C., Sciortino A., Messina F., Buscarino G., Cannas M., Roscigno G., Condorelli G., Cavallaro G., Giammona G., Mauro N. (2019). Highly Homogeneous Biotinylated Carbon Nanodots: Red-Emitting Nanoheaters as Theranostic Agents toward Precision Cancer Medicine. ACS Appl. Mater. Interfaces.

[10] Carrasco J.A., Abellán G., Coronado E. (2018). Influence of morphology in the magnetic properties of layered double hydroxides. J. Mater. Chem. C.

[11] Ansy K.M., Lee J.-H., Piao H., Choi G., Choy J.-H. (2018). Stabilization of antioxidant gallate in layered double hydroxide by exfoliation and reassembling reaction. Solid State Sci..

[12] Yang W., Xia Y., Liu X., Yang J., Liu Y. (2018). Layered double hydroxides/reduced graphene oxide nanocomposites with enhanced barrier properties. Polym. Compos..

[13] Xu Z.P., Gu Z., Cheng X., Rasoul F., Whittaker A.K., Lu G.Q.M. (2011). Controlled release of ketorolac through nanocomposite films of hydrogel and LDH nanoparticles. J. Nanopart. Res..

[14] Mahkam M., Davatgar M., Rezvani Z., Nejati K. (2013). Preparation of pH-Sensitive Polymers/Layered Double Hydroxide Hybrid Beads for Controlled Release of Insulin. Int. J. Polym. Mater. Polym. Biomater..

[15] Lai Y., Li K., Qiao G., Wang F., Zhao H., Yang W., Yang J., Du H. (2018). Structure and magnetic properties of layered compounds RMn1.7Cr0.3Si2C (R = Nd, Sm, Dy). J. Alloys Compd..

[16] Hajibeygi M., Omidi-Ghallemohamadi M. (2018). Preparation and characterization of poly(amide-imide)/Mg-Al LDH nanocomposites; effect of organo-modified LDH on thermal properties and morphology. Polym. Compos..

[17] Ma B., Fernandez-Martinez A., Grangeon S., Tournassat C., Findling N., Carrero S., Tisserand D., Bureau S., Elkaïm E., Marini C. (2018). Selenite Uptake by Ca–Al LDH: A Description of Intercalated Anion Coordination Geometries. Environ. Sci. Technol..

[18] Luo S., Hao J., Gao Y., Liu D., Cai Q., Yang X. (2019). Pore size effect on adsorption and release of metoprolol tartrate in mesoporous silica: Experimental and molecular simulation studies. Mater. Sci. Eng. C.

[19] Sun R., Zhang P., Bajnóczi É.G., Neagu A., Tai C.-W., Persson I., Strømme M., Cheung O. (2018). Amorphous Calcium Carbonate Constructed from Nanoparticle Aggregates with Unprecedented Surface Area and Mesoporosity. ACS Appl. Mater. Interfaces.

[20] Wilczewska A.Z., Niemirowicz K., Markiewicz K.H., Car H. (2012). Nanoparticles as drug delivery systems. Pharmacol. Rep..

[21] Benito P., Guinea I., Herrero M., Labajos F.M., Rives V. (2007). Incidence of Microwave Hydrothermal Treatments on the Crystallinity Properties of Hydrotalcite-like Compounds. Z. Anorg. Allg. Chem..

[22] Yu J., Wang Q., O’Hare D., Sun L. (2017). Preparation of two dimensional layered double hydroxide nanosheets and their applications. Chem. Soc. Rev..

[23] Mishra G., Dash B., Pandey S., Mohanty P.P. (2013). Antibacterial actions of silver nanoparticles incorporated Zn–Al layered double hydroxide and its spinel. J. Environ. Chem. Eng..

[24] Ay A.N., Zümreoglu-Karan B., Temel A., Mafra L. (2011). Layered double hydroxides with interlayer borate anions: A critical evaluation of synthesis methodology and pH-independent orientations in nano-galleries. Appl. Clay Sci..

[25] Mandal S., Tripathy S., Padhi T., Sahu M.K., Patel R.K. (2013). Removal efficiency of fluoride by novel Mg-Cr-Cl layered double hydroxide by batch process from water. J. Environ. Sci..

[26] Wang L.Y., Tong D.S., Zhao L.Z., Liu F.G., An N., Yu W.H., Zhou C.H. (2014). Utilization of alum sludge for producing aluminum hydroxide and layered double hydroxide. Ceram. Int..

[27] Zhao Y., He S., Wei M., Evans D.G., Duan X. (2010). Hierarchical films of layered double hydroxides by using a sol–gel process and their high adaptability in water treatment. Chem. Commun..

[28] Chen C., Gunawan P., Xu R. (2011). Self-assembled Fe_3_O_4_-layered double hydroxide colloidal nanohybrids with excellent performance for treatment of organic dyes in water. J. Mater. Chem..

[29] Gong J., Liu T., Wang X., Hu X., Zhang L. (2011). Efficient Removal of Heavy Metal Ions from Aqueous Systems with the Assembly of Anisotropic Layered Double Hydroxide Nanocrystals@Carbon Nanosphere. Environ. Sci. Technol..

[30] Yu X.-Y., Luo T., Jia Y., Xu R.-X., Gao C., Zhang Y.-X., Liu J.-H., Huang X.-J. (2012). Three-dimensional hierarchical flower-like Mg–Al-layered double hydroxides: Highly efficient adsorbents for As(V) and Cr(VI) removal. Nanoscale.

[31] Xu Y., Kominami K., Ishikawa Y., Feng Q. (2012). Layered hydroxide nickel benzoates: Hydrothermal synthesis, structure characterization, and exfoliation reaction. J. Colloid Interface Sci..

[32] Zhao L., Miao J., Iwasa Y., Feng Q. (2008). Transformation of layered hydroxide zinc benzoate nanosheets into ZnO nanocrystals by electron beam irradiation. J. Ceram. Soc. Jpn..

[33] Quites F.J., Germino J.C., da Silva Azevedo C.K., Moreto J.A., Faleiros M.M., Atvars T.D.Z. (2017). Exfoliation of zinc-layered hydroxide by luminescent conjugate polyelectrolyte: Synthesis and photophysical aspects. J. Sol-Gel Sci. Technol..

[34] Hussein M.Z., Hashim N., Yahaya A.H., Zainal Z. (2010). Synthesis and characterization of [4-(2,4-dichlorophenoxybutyrate)-zinc layered hydroxide] nanohybrid. Solid State Sci..

[35] Zhao L., Wang H., Wang Y., Miao J., Feng Q. (2011). Synthesis of Layered Hydroxide Zinc m-Aminobenzoate Compounds and Their Exfoliation Reactions. Chin. J. Chem..

[36] Chen C., Wang P., Lim T.-T., Liu L., Liu S., Xu R. (2013). A facile synthesis of monodispersed hierarchical layered double hydroxide on silica spheres for efficient removal of pharmaceuticals from water. J. Mater. Chem. A.

[37] Chen C., Felton R., Buffet J.-C., O’Hare D. (2015). Core–shell SiO_2_@LDHs with tuneable size, composition and morphology. Chem. Commun..

[38] Zhu R., Wang Z., Liang P., He X., Zhuang X., Huang R., Wang M., Wang Q., Qian Y., Wang S. (2017). Efficient VEGF targeting delivery of DOX using Bevacizumab conjugated SiO_2_@LDH for anti-neuroblastoma therapy. Acta Biomater..

[39] Mallakpour S., Naghdi M. (2018). Polymer/SiO_2_ nanocomposites: Production and applications. Prog. Mater. Sci..

[40] Zou H., Wu S., Shen J. (2008). Polymer/Silica Nanocomposites: Preparation, Characterization, Properties, and Applications. Chem. Rev..

[41] Nakabayashi H., Yamada A., Noba M., Kobayashi Y., Konno M., Nagao D. (2010). Electrolyte-Added One-Pot Synthesis for Producing Monodisperse, Micrometer-Sized Silica Particles up to 7 μm. Langmuir.

[42] Zhu W., Li X., Wu D., Yu J., Zhou Y., Luo Y., Wei K., Ma W. (2016). Synthesis of spherical mesoporous silica materials by pseudomorphic transformation of silica fume and its Pb^2+^ removal properties. Microporous Mesoporous Mater..

[43] Cao C., Li Z., Li Y., Wang G., Yuan S., Li H., Guo P., Zhao X.S. (2017). Synthesis, characterization and electrochemical applications of Ir@SiO_2_ composite microspheres. Colloids Surf. A: Physicochem. Eng. Asp..

[44] Alimunnisa J., Ravichandran K., Meena K.S. (2017). Synthesis and characterization of Ag@SiO_2_ core-shell nanoparticles for antibacterial and environmental applications. J. Mol. Liq..

[45] Zhao B., Tian C., Zhang Y., Tang T., Wang F. (2011). Size control of monodisperse nonporous silica particles by seed particle growth. Particuology.

[46] Ma Q., Li Z.R., Niu H.W., Wang Z.Y., Ba J., Qi J.L., Feng J.C., He P., Ma J. (2018). The effect of crystal structure of SiO_2_ on the wettability of AgCuTiSiO_2_f/SiO_2_ system. Vacuum.

[47] Haraketi M., Hosni K., Srasra E. (2017). Intercalation behavior of salicylic acid into calcined Cu-Al-layered double hydroxides for a controlled release formulation. Surf. Eng. Appl. Electrochem..

[48] Pooresmaeil M., Behzadi Nia S., Namazi H. (2019). Green encapsulation of LDH(Zn/Al)-5-Fu with carboxymethyl cellulose biopolymer; new nanovehicle for oral colorectal cancer treatment. Int. J. Biol. Macromol..

[49] Miao J., Xue M., Itoh H., Feng Q. (2006). Hydrothermal synthesis of layered hydroxide zinc benzoate compounds and their exfoliation reactions. J. Mater. Chem..

[50] Yue Y., Wang J., Zhang Y., Song Y., Zuo X. (2018). Interactions of atomic hydrogen with amorphous SiO_2_. Phys. B Condens. Matter.

[51] Tolnai G., Csempesz F., Kabai-Faix M., Kálmán E., Keresztes Z., Kovács A.L., Ramsden J.J., Hórvölgyi Z. (2001). Preparation and Characterization of Surface-Modified Silica-Nanoparticles. Langmuir.

[52] Godjevargova T., Velikova M., Vasileva N., Dimova N., Damyanov D. (2005). Metallochelate immobilization of urease on to amorphous SiO_2_. Process Biochem..

