# Supplementary material for: A Facile Synthesis of Core-Shell SiO2@Cu-LBMS Nano-Microspheres for Drug Sustained Release Systems
Source: Materials (Basel). 2019 Nov 30;12(23):3978. doi: 10.3390/ma12233978 (PMC6926544; doi:10.3390/ma12233978)
Supplement: Supplementary file 1 [file materials-12-03978-s001.pdf]

materials

Supplementary Materials

A Facile Synthesis of Core-shell SiO_2@Cu-LBMS Nano-Microspheres for Drug Sustained Release System

Hui Wang [1], Haifeng Yang [2]* and Lifang Zhao [1]

[1] College of Chemistry and Chemical Engineering, Shaanxi Key Laboratory of Phytochemistry, Baoji University of Arts and Sciences, Baoji, Shaanxi, 721013, China; wangkejian@163.com (H.W); zlfzhao@163.com (L.Z)

[2] College of Physics and Optoelectronics Technology, Medical Micro-nano Materials Research Center, Baoji University of Arts and Sciences, Baoji 721016, China

* Correspondence: yanghaifeng@bjwlxy.edu.cn

Supplementary Materials

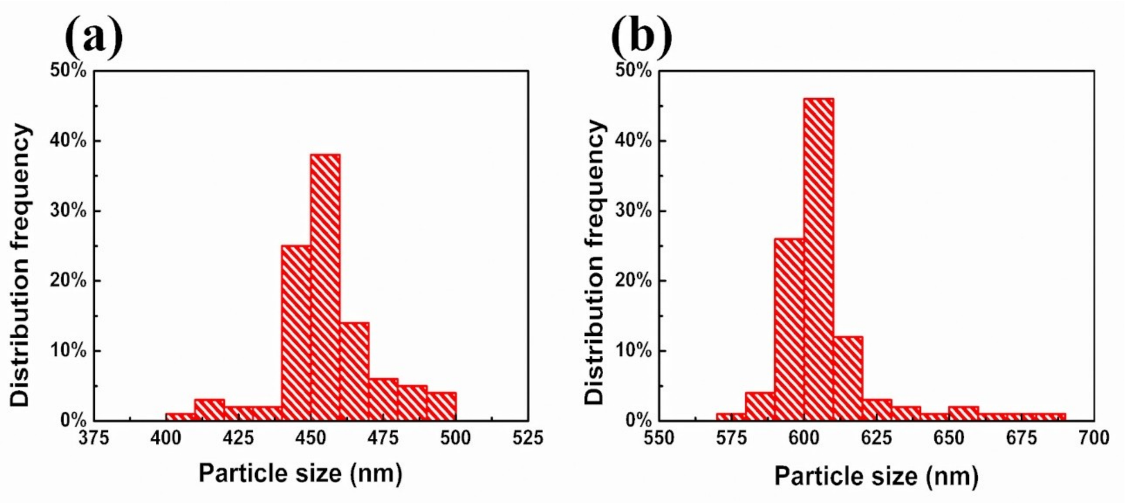

Figure S1. Particle size distribution of (a) SiO_2; (b) SiO_2@Cu-LBMS.

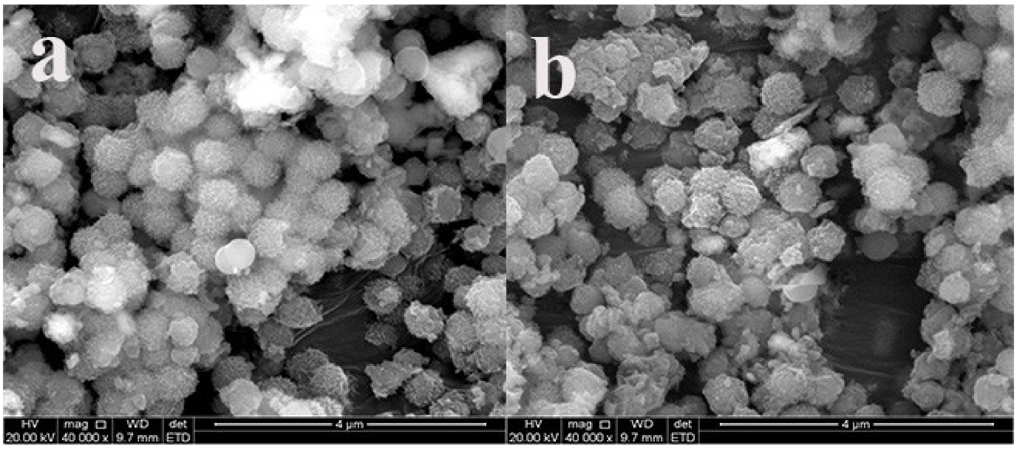

Figure S2. SEM micrographs of SiO_2@Cu-LBMS with different reaction time (a) 19h; (b) 37h

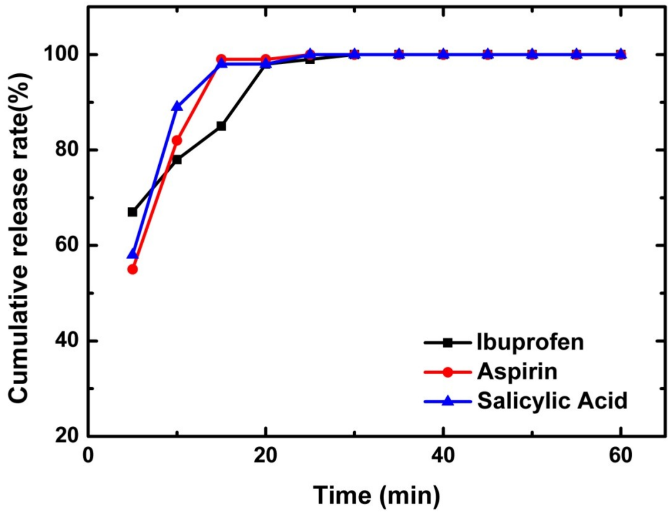

Figure S3. Cumulative release rate of physical mixture

Table S1. Drug cumulative release rate (%) of Cu-LBMS and SiO_2@Cu-LBMS.

Time(h)	Cu-LBMS			SiO_2@Cu-LBMS		
	Ibuprofen (%)	Aspirin (%)	Salicylic Acid (%)	Ibuprofen (%)	Aspirin (%)	Salicylic Acid (%)
0.5	25	17.2	14.5	8.3	7.8	7.7
1	30.6	40.1	36.5	13.3	11.5	15.3
1.5	40.1	56.3	58.3	19.7	15.4	18.6
2	45.6	74.9	74.2	22.7	24.3	25.4
2.5	58.2	79	84.5	26.7	33.1	34.3
3	70.8	82.9	87.3	35.3	36.3	38.3
3.5	81.4	86.8	87.2	40.2	48.1	42.4
4	89.5	90.7	87.4	41.4	52.4	56.5
4.5	91.2	90.7	87.3	55.6	60.3	70.3
5	91.3	90.7	87.2	63.2	79.9	80.1
5.5	91.2	90.7	87.3	72.3	82.1	83.5
6	91.3	90.7	87.3	85.1	84.1	89.5
6.5	91.3	90.7	87.3	91.8	89.1	91.5
7	91.3	90.7	87.2	92.3	92.1	91.5
7.5	91.3	90.7	87.3	92.3	94.1	91.5
8	91.3	90.7	87.2	92.3	94.1	91.5

© 2019 by the authors. Submitted for possible open access publication under the terms and conditions of the Creative Commons Attribution (CC BY) license (http://creativecommons.org/licenses/by/4.0/).
